# Outcomes of Strabismus Surgery in Pediatric Hydrocephalus: Does Shunting Influence Surgical Success?

**DOI:** 10.3390/children13081031

**Published:** 2026-08-03

**Authors:** Didem Dizdar Yigit, Sule Nur Kandemir, Volkan Dericioglu, Furkan Cam, Yener Sahin, Eren Cerman, Adnan Dagcinar, Hande Celiker

**Affiliations:** 1 Department of Ophthalmology, Marmara University, 34854 Istanbul, Türkiye; suleekndmr@gmail.com (S.N.K.); volkandr@gmail.com (V.D.); camfurkan25@gmail.com (F.C.); drhandeceliker@yahoo.com (H.C.); 2 Department of Neurosurgery, Marmara University, 34854 Istanbul, Türkiye; yenersah@gmail.com (Y.S.); adnan.dagcinar@marmara.edu.tr (A.D.); 3 Department of Ophthalmology, Klinik Donaustadt, 1220 Vienna, Austria; eren.cerman@gesundheitsverbund.at

**Keywords:** pediatric hydrocephalus, hydrocephalus, ventriculoperitoneal shunt operation, strabismus, strabismus surgery

## Abstract

**Highlights:**

**What are the main findings?**

- Strabismus was common in pediatric patients with hydrocephalus, with esotropia being the predominant deviation pattern and amblyopia occurring more frequently than expected in the general pediatric population.
- In this limited cohort, neurological comorbidities such as spina bifida and epilepsy were more frequent among patients with strabismus, while no clear association was detected between VP shunt status and surgical success.

**What are the implications of the main findings?**

- Pediatric patients with hydrocephalus should undergo regular ophthalmologic follow-up, as strabismus and amblyopia are common and may require early refractive correction, amblyopia treatment, or surgical management.
- Strabismus management in hydrocephalus should be individualized, taking into account neurological comorbidities, systemic stability, shunt status, and developmental factors; when the child is clinically stable and surgery is indicated, acceptable ocular alignment may be achievable.

**Abstract:**

**Background/Objectives**: The primary aim of this study was to evaluate the rate of strabismus and surgical outcomes in pediatric hydrocephalus (HC) patients, and compare the results in patients with and without ventriculoperitoneal (VP) shunt surgery. **Methods**: This study includes pediatric HC patients who were followed at the pediatric ophthalmology and strabismus unit of a tertiary hospital. Detailed ophthalmological examinations including deviation angle were performed. Preoperative measurements and surgeries were carried out by one strabismus expert. Postoperative deviation of ≤10 prism diopters (PD) was considered successful. **Results**: In total, 64 subjects were included in this study (29 female, 35 male), and 42 had a VP shunt (shunt+), while 22 were shunt−. The mean age of participants was 8.5 ± 4.6 years, and 50% (n = 32) of them had strabismus (22 esotropia, 10 exotropia). Both groups had similar refractive error and visual acuities (*p* > 0.05). Amblyopia rates were 21% (9/42) vs. 23% (5/22) in the shunt+ and shunt− groups. In 40% of shunt+ patients (17/42) there was strabismus, compared to 68% of shunt− patients (15/22). Among 22 patients with esotropia, 12 underwent strabismus surgery. Surgical success was achieved in nine patients (75%). In exotropic patients only one had surgery and it was successful. The operation rate in the strabismus group was 41% (13/32). Others mostly did not have surgery due to systemic comorbidities. Patients with strabismus had approximately 3.7 times higher odds of having epilepsy or spina bifida compared to those without strabismus (*p* = 0.049; OR = 3.69; 95% CI: 1.13–12.11). **Conclusions**: These findings suggest that children with hydrocephalus may benefit from regular pediatric ophthalmologic follow-up to detect strabismus, amblyopia, and refractive errors at an early stage. Although acceptable alignment outcomes were observed in selected operated patients, the small surgical cohort limits definitive conclusions regarding surgical success or the influence of VP shunting; therefore, treatment decisions should be individualized within a multidisciplinary framework.

## 1. Introduction

Hydrocephalus (HC) is characterized by an abnormal accumulation of cerebrospinal fluid (CSF) within the cerebral ventricles, arising from disturbances in CSF production, circulation, or absorption, or mechanical obstruction within the ventricular pathways [1]. Congenital malformations represent the most frequent cause in newborns, whereas intraventricular hemorrhage is a predominant etiology among preterm infants [2]. Infections, trauma and intracranial tumors are other etiologies. Strabismus is the most frequent ocular symptom in HC patients. In the literature, the reported incidence of strabismus among pediatric HC cases varies, ranging from 30% to as high as 60–70% [3,4]. Hydrocephalus may disturb ocular alignment through several pathways, including abducens nerve dysfunction related to elevated intracranial pressure, supranuclear gaze and vergence abnormalities, and injury to periventricular/posterior visual pathways. Optic nerve damage and cerebral visual impairment may additionally impair binocular visual development and sensory fusion [3,5].

The sudden onset of strabismus, other ocular motility disorders, or papilledema often indicates uncontrolled intracranial pressure in HC. Although shunt surgery is the initial and most critical intervention, it does not always lead to the restoration of normal ocular motility or visual function [5]. If prematurity accompanies the situation, the risk of strabismus significantly increases after intraventricular hemorrhage [6].

Esotropia is the most frequent form of heterotropia in HC patients [3,7]. The underlying mechanism is not clear, but it is suggested that sixth nerve palsy due to high intracranial pressure and high refractive errors might be the potential causes [3,7]. Not all children with HC and strabismus require surgery. In one clinical series, only 5 out of 17 children diagnosed with both HC and strabismus underwent surgical intervention, while the remainder were managed with glasses or follow-up. This represents a surgical intervention rate of approximately 30%. The decision to proceed with surgery typically depends on the severity and type of strabismus, systemic comorbidities and whether it can be adequately corrected with optical treatment [7].

Evidence regarding strabismus surgery in children with hydrocephalus or other neurological disorders remains limited. In a retrospective series of 17 children with hydrocephalus and strabismus, only 5 underwent surgery, of whom 4 achieved satisfactory ocular alignment after a single procedure [7]. Similarly, Swaminathan et al. reported successful alignment in 60% of children with developmental delay compared with 74% of typically developing controls; however, the developmental delay group received a reduced surgical dose and postoperative follow-up was limited to six weeks [8]. The available evidence is based on small and heterogeneous cohorts, and the optimal surgical dose and prognostic factors remain uncertain.

In pediatric HC patients, several central nervous system (CNS) problems like spina bifida and epilepsy might be observed as a comorbidity. The rate of spina bifida in pediatric HC patients is approximately one out of five cases and stands out as a prominent cause of congenital HC [9]. Most studies report that epilepsy develops in about 20–30% of children with HC [10]. CNS comorbidities in HC patients might negatively affect the visual prognosis as they are also common causes of cerebral visual impairment (CVI) [11,12]. HC patients with epilepsy was reported to have more ocular pathologies like strabismus, nystagmus, and amblyopia compared to their age- and sex-matched peers [3].

The primary aim of this study was to evaluate the rate of strabismus and surgical outcomes in pediatric HC patients, and compare the results in patients with and without shunt surgery.

## 2. Materials and Methods

This is a retrospective study including 64 pediatric HC patients (29 females, 35 males). The subjects were selected from pediatric ophthalmology and strabismus unit of a tertiary hospital. The Institutional Review Board (IRB) approved this study as it adhered to the ethical tenets of the Declaration of Helsinki. Data acquisition and analysis were performed in compliance with the protocols approved by the Ethical Committee of Marmara University (Protocol no: 09.2024.1521). An informed consent to participate was obtained from the parents/legal guardians of the patients.

Inclusion criteria were being under 18 years old and being diagnosed with HC. Exclusion criteria were missing data in patients’ files. HC patients who had undergone shunt placement were categorized as shunt-positive (shunt+), while those without a shunt were categorized as shunt-negative (shunt−). All patients who underwent shunt surgery had their operation in our University Neurosurgery Clinic. Ophthalmologic examination was performed after neurosurgical stabilization; all shunt-positive patients had stable intracranial pressure during the ophthalmologic evaluation period, and none required shunt revision for dysfunction at that time.

All subjects were assessed with detailed ophthalmological examination including deviation angle measurements with prisms. Refractive errors were measured by autorefractometry [Nidek ARK-530 (Gamagori, Aichi, Japan) when possible. Two drops of cyclopentolate (0.5% for children under 1 year old, 1% for those who were over 1 year old) were administered 10 min apart. Cycloplegic refraction was performed in all cooperative patients. Distance visual acuity (VA) was measured and noted in decimals with tumbling “E” optotypes in children over 2 years old. In testing distance VA for children below 2 years old and who were not receptive to tumbling E’s, “fix and follow” (FF) ability was noted as positive or negative. Amblyopia was reported as the cause of visual loss with no apparent organic lesion. Because formal CVI testing was not available in all patients, amblyopia was defined clinically based on asymmetric fixation/following response, age-inappropriate VA, or an interocular difference of at least two lines in the absence of an apparent ocular structural lesion. We acknowledge that differentiation from CVI may be challenging in neurologically impaired children [13].

Deviation measurements were performed by prism occlusion test or Krimsky, according to the patient’s fixation status. Deviation angles were measured at distance and near fixation. Detailed systemic and ophthalmological history of patients were obtained from their records. Surgical criteria were a stable manifest deviation not adequately corrected by refractive treatment, sufficient systemic and neurological stability for anesthesia and follow-up, and a clinically meaningful functional or psychosocial indication. All strabismus surgeries were performed under the supervision of one strabismus specialist. Surgical success was assessed at the last postoperative visit. A postoperative deviation of ≤10 prism diopters (PD) was considered a successful outcome.

As this was a retrospective study, no a priori sample size calculation was performed. All consecutive patients who met the eligibility criteria during the study period were included.

Based on the observed difference in associated CNS pathology between patients with and without strabismus, approximately 49 patients per group would be required to detect a comparable difference with 80% power and a two-sided alpha level of 0.05 in a future multicenter study, according to calculations made by using the G Power tool.

### Statistical Evaluation

Descriptive statistics were given as mean, standard deviation, median, minimum, maximum, percentage, and ratio values where relevant. The distribution of data was analyzed with the Shapiro–Wilk test. Parametric or non-parametric tests were used according to the distribution of data. Comparative analyses were performed to evaluate differences in clinical and surgical outcomes between the two groups of shunt+ and shunt−. Continuous variables were compared using the independent samples *t*-test or Mann–Whitney U test, as appropriate. Categorical variables were analyzed using the Chi-Square test or Fisher’s exact test when expected frequencies were <5. A *p*-value less than 0.05 was considered statistically significant. Odds ratios (OR) with corresponding 95% confidence intervals were calculated to assess the strength of association between binary categorical variables. Fisher’s exact test was used to compare the presence of associated CNS pathology between the strabismus and non-strabismus groups. As this analysis involved a single predefined comparison, no adjustment for multiple testing was applied. The analysis was made using SPSS program version 28.0 (SPSS Inc, Chicago, IL, USA).

## 3. Results

A total of 64 subjects with HC were included in this study (32 with strabismus, 32 orthotropic). The mean age of the 64 patients (29 female, 35 male) was 8.5 ± 4.6 years, and 42 patients were shunt+, while 22 were shunt−. Both groups had similar refractive error and visual acuities (*p* > 0.05). The refractive errors were 1.30 ± 2.44 diopters (D) and 1.25 ± 2.56 D in the shunt+ and shunt− groups, respectively. The VA was tested with the FF test in 54% (12/22) of shunt− patients, while the FF test was performed in 40% of shunt+ patients. The rest of the subjects were tested with tumbling E’s, and the mean VA was 0.52 ± 0.33 and 0.51 ± 0.33 (*p* > 0.05) in the shunt+ and shunt− groups, respectively (Table 1). Amblyopia rates were 21% (9/42) vs. 23% (5/22) in the shunt+ and shunt− groups. Six of them were strabismic, and eight of them had either anisometropia or high refractive error (more than +5.0 D hyperopia, −4.0 D myopia, ±2.0 D astigmatism).

In the strabismus group, 22 patients had esotropia of 33.68 ± 10.76 PD and 10 had exotropia of 34.4 ± 11.68 PD. In 40% of shunt+ patients (17/42) there was strabismus, compared to 68% of shunt− patients (15/22). Although Fisher’s exact test did not reach statistical significance (*p* = 0.064), the data show a trend suggesting that shunt presence was associated with lower odds of strabismus (OR = 0.32, 95% CI: 0.11–0.94). A non-significant trend toward lower strabismus prevalence among shunt-positive patients was observed, but this finding should not be interpreted as evidence of a protective effect.

Among 22 patients with esotropia, 12 underwent strabismus surgery. All of them had bimedial rectus recession surgery. The mean deviation angle of the patients was 3.80 ± 6.5 PD postoperatively (Table 2). Surgical success was achieved in nine patients (75%) and failure in three patients (25%). One of these three patients had sixth nerve palsy, one had spina bifida and the other had a history of intraventricular hemorrhage. Of those with a shunt, four out of seven surgeries were successful (57%), whereas six out of six patients without a shunt achieved successful alignment (100%). In exotropic patients only one had bilateral lateral rectus recession surgery and it was successful. The average postoperative follow-up duration was 19.1 ± 3.1 months. The operation rate in the strabismus group was 41% (13/32). The other patients mostly did not have surgery due to systemic comorbidities.

The HC etiology was primary congenital HC in 19 of the patients (30%). Meanwhile, 15 patients had myelomeningocele or spina bifida (23%). Other rare causes were meningitis (3%), Dandy–Walker Syndrome (3%), intracranial tumors (3%), and pathologies related to prematurity. In the strabismus group, 14 had a history of prematurity (44%), while in the orthophoric group only 7 were premature (16%) (*p* > 0.05). Three of the premature patients had a history of intraventricular hemorrhage with shunt operation and strabismus.

Among 64 HC patients, CNS pathologies like spina bifida and epilepsy was observed in 13 of 32 patients with strabismus (40.6%) and in 5 of 32 patients without strabismus (15.6%). Fisher’s exact test revealed a statistically significant association between strabismus and CNS pathology (*p* = 0.049) (Table 3). Patients with strabismus had approximately 3.7 times higher odds of having epilepsy or spina bifida compared to those without strabismus (OR = 3.69; 95% CI: 1.13–12.11).

When the shunt revision rates were analyzed, in the strabismus group, 12 had shunt revisions out of 21 shunt+ patients (57%), whereas 14 patients had shunt revision operations out of 21 shunt+ in the non-strabismus group (66%) (*p* > 0.05). The amblyopia rate and surgery success were not found to be correlated with shunt revisions.

## 4. Discussion

In pediatric HC patients, strabismus is the most frequent ophthalmological sign. In this study the strabismus rate was 50% in pediatric HC patients. In the literature the rates show variability between 33 and 70% [4,5,14]. Esotropia is the most frequent form of heterotropia in HC patients [3,7]. In our cohort it was also the case. Among strabismic subjects, 69% (22/32) had esotropia.

It is not clear if having shunt surgery would be protective. The data in the literature are highly variable, and some reports claim that successful control of intracranial pressure might correct ocular alignment. AlObaisi et al. reported that in 33.1% of 190 children with shunted HC, strabismus was also present. They found a statistically significant correlation between VP shunt and strabismus [15]. This discrepancy with our results may be explained by differences in patient populations, hydrocephalus etiologies, disease severity, and inclusion criteria between the two studies. In addition, the relatively small sample size of our cohort may have limited the statistical power to detect similar associations. Aring et al. also reported that ocular anomalies were detected in 69% of shunted meningomyelocele patients who had VP shunt operation before 1 year of age [3]. On the other hand, there are cases with corrected ocular alignment after timely correction of intracranial pressure with a shunt operation [16]. Although the observed proportions differed, the comparison was not statistically significant; therefore, no protective effect of shunting can be inferred from our dataset. If the optic atrophy or pressure remains high during a longer period, the shunt might not have any effect on ophthalmological symptoms [17].

Success of strabismus surgery was found similar to pediatric strabismus surgery results in the literature. In a series of 17 pediatric patients, 4 out of 5 patients (80%) who had strabismus surgery had good ocular alignment after single surgery [7]. Other patients were managed conservatively with refractive error correction. Another study giving strabismus results in meningomyelocele and HC patients reported the rate of excellent/satisfactory results as 73% [18]. In the presented study, the overall surgical success rate after strabismus surgery was 77%. This rate is comparable to non-HC pediatric strabismus surgery results. The observed alignment rate was within the range reported in previous pediatric strabismus series; however, comparisons with non-hydrocephalic populations should be interpreted cautiously because of the small and selected surgical subgroup.

Pediatric HC patients with strabismus might have other systemic problems like prematurity and CNS pathologies. Most common accompanying pathologies are spina bifida and epilepsy. Both HC and epilepsy are common causes of CVI, which is defined as the visual disturbance due to retrogeniculate pathway damage. In CVI, the ocular structures are intact or the vision loss is greater than the existing ocular pathology [11,12]. HC might affect vision both by causing optic nerve damage through pressure changes and by causing CVI by damaging posterior pathways close to lateral ventricles. There is no established therapy for CVI, but it is emphasized that the damage might be reversible and early visual stimulation is encouraged [19].

Even if the results might be unpredictable, in order to have accurate visual stimulation in early visual developmental stages, children with developmental delay and large esotropia were indicated for surgery with some under-correction [8]. In this cohort, most of the patients were observed with glasses and follow-up (80%), while in the operated group (20%), the surgical success was 77% (10/13). The correction of ocular alignment might have a positive effect on visual prognosis. The amblyopia rates were similar in the shunt+ and shunt− groups. However, amblyopia rate in this pediatric HC population (20%) was higher than the general population aged 0–19 years (1–3%) [20]. Half of them were strabismic and half of them had significant refractive errors. Altıntaş et al. also reported that the risk of amblyopia due to strabismus or significant refraction error was 56% in their pediatric HC population [4]. Since these children are at risk of both CVI and amblyopia, modifiable factors such as refractive error or strabismus correction might be crucial for achieving better visual results.

Prematurity is another topic to be considered in these children because it also adds the risk of ophthalmological problems like strabismus, high refractive errors, and amblyopia. If prematurity accompanies the situation, the risk of strabismus significantly increases due to other coexisting systemic problems [6]. In this sample, the prematurity rate has a trend to be higher among the strabismus group compared to the non-strabismus group (44% vs. 16%), but the difference was not statistically significant due to the small sample size. Advances in neonatal intensive care units have improved survival rates, resulting in a higher number of preterm infants surviving. These children are encountered more frequently in strabismus clinics, so prematurity should also be noted.

HC has a variety of etiologies like congenital, trauma, tumor, etc. This study evaluated patients according to etiologies and systemic conditions like prematurity or CNS abnormalities while giving ophthalmological features. The management of these strabismic HC patients should include multiple factors including systemic condition. These patients are at risk of intracranial pressure imbalances, and need shunt or shunt dysfunction/revision. This means ocular alignment might be affected at any given time of the process (even after strabismus surgery). All of the patients had stabilized intracranial pressure at the time of ophthalmological examination and none of them were assigned to have a shunt operation/revision due to dysfunction. This was crucial because shunt dysfunctions and revisions might have an effect on the pathological process due to intracranial pressure changes. In a study by Altıntaş et al., the number of shunt revisions were found to be associated with strabismus and amblyopia; however, others suggested that this correlation is weak [3,4]. Shunt dysfunctions were previously correlated with ophthalmological signs like strabismus, papilledema and amblyopia along with CVI [21,22]. In the present study, shunt revisions were not found to be correlated with amblyopia, strabismus or surgery outcomes. This might be due to differences between study samples, timely intervention and the experience of the neurosurgeon or emerging technologies in neurosurgery in the last decades [23].

This study has several limitations. First, its retrospective design and relatively small sample size, particularly the limited number of operated patients, restrict the strength of conclusions regarding surgical outcomes and the influence of VP shunting. In addition, objective indicators of HC severity, including ventricular size, intracranial pressure parameters, and radiological severity grading, were not available in this cohort; therefore, their potential influence on strabismus development and surgical outcomes could not be assessed. Furthermore, potential confounders such as prematurity, neurological comorbidities, HC etiology, shunt revision history, VA, refractive error, and amblyopia could not be fully controlled with multivariable analysis. Formal CVI assessment was not performed, and therefore the contribution of CVI to reduced visual function could not be quantified. Comparisons between VP-shunted and non-shunted patients should be interpreted with caution, as these groups may differ in terms of hydrocephalus etiology, disease severity, neurological status, timing of diagnosis, and other clinical characteristics that were not uniformly available. These factors may have introduced residual confounding and selection bias. The operated subgroup may not represent all strabismic hydrocephalus patients, because surgical candidacy was influenced by systemic condition, cooperation, neurological stability, and feasibility of postoperative follow-up.

A complete ophthalmic examination should be part of the evaluation of all pediatric HC patients to identify and treat ophthalmologic problems at an early stage. Strabismus, in particular, should be closely monitored and treated when the systemic condition of the child permits. Regular ophthalmic supervision by a pediatric ophthalmologist might provide and help to maintain the best possible results in children with HC. The collaboration of neurosurgeons, neuro-ophthalmologists and pediatric ophthalmologists might detect and address common signs in HC patients.

## 5. Conclusions

To conclude, in this limited retrospective cohort, strabismus was common among pediatric patients with hydrocephalus. When surgery was feasible, acceptable alignment was observed in selected patients; however, the small surgical subgroup precludes firm conclusions regarding the effect of shunt status on surgical success. Also it might be recommended to correct refractive errors or perform strabismus surgery when possible, because amblyopia rate in these children is higher and any measure should be taken in order to have better visual prognosis. These findings should be considered exploratory and support larger multicenter studies with standardized CVI assessment and adjustment for neurological severity.

## Figures and Tables

**Table 1:** Participants’ characteristics.

	Shunt+ (n = 42)	Shunt− (n = 22)
**Mean age (years)**	8.02 ± 4.40	8.73 ± 4.49
**Mean refractive error (D)**	1.30 ± 2.44	1.25 ± 2.56
**Mean visual acuity**	0.52 ± 0.33	0.51 ± 0.33
**Strabismus prevalence**	40% (17/42) CI, 25.6–56.7%	68% (17/22) CI, 45.1–86.1%

**Table 2:** Preoperative and postoperative deviation angles in strabismic patients.

	Esotropia (n = 22)	Exotropia (n = 10)
**Mean deviation angle (PD)**	33.68 ± 10.76	34.4 ± 11.68
**Surgeries performed**	12	1
**Postoperative deviation angle (PD)**	3.80 ± 6.5	0

**Table 3:** CNS pathology rates in strabismic and non-strabismus patients.

	Strabismus (n = 32)	Non-Strabismus (n = 32)	*p*	OR (%95 CI)
CNS pathology (spina bifida or epilepsy)	13 (40.6%)	5 (15.6%)	0.049 *	3.69 (1.13–12.11)
No CNS pathology	19 (59.4%)	27 (84.4%)	0.049 *	

* Fisher’s exact test. CI: confidence interval. CNS: central nervous system. OR: odds ratio.

## Data Availability

The data presented in this study are available on request from the corresponding author due to privacy, ethical reasons.

## Abbreviations

The following abbreviations are used in this manuscript:

CVI	Cerebral visual impairment
HC	Hydrocephalus
VA	Visual acuity
VP	Ventriculoperitoneal
